# Balancing Work and Cancer Care: Challenges Faced by Employed Informal Caregivers

**DOI:** 10.3390/cancers14174146

**Published:** 2022-08-27

**Authors:** Ellen Xiang, Patricia Guzman, Martha Mims, Hoda Badr

**Affiliations:** 1 Department of Medicine, Section of Epidemiology and Population Science, Baylor College of Medicine, Houston, TX 77030, USA; 2 McGovern Medical School, Health Science Center, The University of Texas, Houston, TX 77225, USA; 3 Department of Medicine, Section of Hematology & Oncology, Baylor College of Medicine, Houston, TX 77030, USA

**Keywords:** caregivers, cancer, employment, narrative review

## Abstract

**Simple Summary:**

Caregiving for a family member or close friend with cancer can be particularly demanding for employed individuals who are juggling work responsibilities while providing care. With an eye toward stimulating research to develop programs and resources to support this vulnerable subgroup of caregivers, this narrative review first describes the financial, work, and mental health impacts of cancer on employed caregivers. Next, critical knowledge gaps are identified and directions for future research are described. The article concludes by formulating an agenda for practice that includes a multipronged effort on behalf of employers, healthcare, and community-based organizations to support and empower employed cancer caregivers.

**Abstract:**

Individuals with cancer commonly rely on their informal caregivers (e.g., spouse/partner, family member, close friend) to help them manage the demands of the disease and its treatment. Caregiving, including helping with patient care, performing household chores, and providing emotional and practical support, can be particularly demanding for employed caregivers, who must juggle their work responsibilities while providing care. Although a burgeoning literature describes the toll that balancing these oft-competing demands can exact, few resources exist to support employed cancer caregivers. To address this gap, we conducted a narrative review of the impacts of cancer on employed caregivers. We found that employed caregivers experience significant financial impacts in terms of lost time and income. They also experience a variety of work-related (e.g., reduced productivity, absenteeism) and mental health (e.g., stress, burden) impacts. Going forward, prospective studies are needed to characterize changes in caregiver support needs and preferences at different time points along the cancer care continuum (e.g., at diagnosis, during treatment, end-of-life) so that appropriate workplace accommodations can be provided. More population-based studies are also needed to develop models for identifying caregivers who are at increased risk for poor employment or mental health outcomes so that more targeted support programs can be developed. Ultimately, a multipronged effort on behalf of employers, healthcare, and community-based organizations may be needed to support and empower this vulnerable subgroup.

## 1. Introduction

There are an estimated 53 million adults in the United States (U.S.) who are providing unpaid care and support to a spouse, partner, close family member, or friend with a chronic or serious health condition [1]. These informal caregivers have been described as a national resource because they provide a significant portion of outpatient care, reducing burden on the healthcare system [2]. Although the proportion of informal caregivers for individuals with cancer is estimated as 7–15% of the total caregiver population [1,3], their experience is often characterized as distinct from those caring for individuals with dementia or other chronic conditions due to the abrupt onset and variable trajectory of the disease [4]. Informal cancer caregivers provide a broad range of essential services including assistance with activities of daily living, symptom management, social and emotional support, and advocacy (e.g., dealing with healthcare providers and insurance) [5,6,7,8,9]. Moreover, the growing reliance on outpatient care in oncology has resulted in informal caregivers performing increasingly complex care tasks—often with little training or support [7,10]. Thanks to advances in early detection and treatment, more individuals are also living longer after a cancer diagnosis [11]. As a result, there is a growing population of survivors who will spend their lives coping with the long-term and late effects of cancer and its treatment [12]. This extends the burden placed on informal caregivers [13,14]—half of whom are also employed [15]. It is estimated that cancer caregivers spend an average of 32.9 h per week providing care, with 32% providing >40 h of care per week, which is comparable to having a full-time job [16]. Over the past two decades, a burgeoning literature describing the toll that juggling work and caregiving responsibilities can exact on caregivers has emerged, yet few resources currently exist to support caregivers in navigating and coping with these demands. Therefore, with an eye toward stimulating research to develop programs and resources to support this vulnerable subgroup, this narrative review seeks to first describe the current state of the science on the impact of cancer on employed caregivers. Rather than addressing a specific research question, as are the aims of other reviews including systematic reviews, this narrative review will provide a broad overview and synthesis of the literature in this emerging field. Based on this, some key knowledge gaps are identified and an agenda for future research and practice is articulated.

## 2. Costs of Care

Estimates suggest that informal caregiving accounts for 18–33% of the total cost of cancer care [17]. The economic value of the services caregivers provide has been calculated using different methods [18]. The opportunity cost method assesses the value of what caregivers give up when they provide care. This is usually based on the caregivers’ lost wages, but it can also include estimates derived from other techniques (e.g., conjoint analysis, estimates of the monetary value of travel time or lost leisure time). The replacement cost (or proxy good) method assesses time spent on informal care through the labor market prices of a close market substitute (e.g., a housekeeper for housekeeping services, and a nurse for healthcare services).

One U.S. study that used the opportunity cost method to estimate the economic burden of localized prostate cancer found that the mean annual economic burden to caregiving partners was $6063 (Range $571–$47,105) with the wide variation attributed to patient and caregiver characteristics [19]. A UK study using this method estimated the cost of informal care to cancer patients at end-of-life to be £948.86 per week, with social/emotional support and symptom management tasks representing the largest proportion of the monetary valuation [20].

Studies using the proxy good method have estimated the value of informal care as ranging widely from $975 to $19,112 per month (Mean = $4809 per month, SD = $6441) [18]. With regard to cancer type, lung cancer appears to have the highest cost of informal care ($4784 per month), followed by ovarian cancer ($4357 per month), with breast cancer caregivers incurring the lowest care cost ($2523 per month) [21]. With regard to phase, two Irish studies compared the cost of informal care during the initial cancer diagnosis and treatment phases and found that cost was higher for the initial diagnosis phase, suggesting that caregivers may have had to take more time off from work during this period (e.g., to take the patient for tests or second opinions) [22,23]. Overall, however, studies suggest that terminal cancer patients have the highest informal care costs [24,25].

Beyond this, cancer caregiving has been associated with out-of-pocket costs. Neglecting to account for out-of-pocket costs could lead to underestimation of the true cost of informal cancer caregiving as estimates suggest they make up 7–13% of total caregiver costs [22,24]. Types of out-of-pocket costs include insurance deductibles and co-pays for healthcare and medications, nutritional supplements and meals at the hospital, parking and travel costs for medical appoints, and formal help (e.g., professional care or paid domestic help) [26]. These costs range widely across studies (M = $447 per month, SD = $394, Range $25–$1233)—largely due to differences in the types of costs that are assessed and the phase/type of cancer that is examined [18].

## 3. Work-Related Impacts of Cancer Caregiving

The work-related impacts of informal cancer caregiving that have been investigated in the literature include: (1) labor market withdrawal to provide care, (2) work modifications, (3) absenteeism, and, (4) presenteeism. Each of these is described below.

### 3.1. Labor Market Withdrawal to Provide Care

In one of the few studies to examine the long-term employment outcomes of cancer caregivers, Veenstra et al. [27], surveyed 240 employed partners of women with early-stage breast cancer and found that 90% were still working four years after the patient’s diagnosis. On the surface, this may appear to suggest that being a caregiver does not result in labor market withdrawal or losing one’s job. Indeed, caregivers may be reluctant to quit their jobs in order to maintain their employer-based health insurance—particularly when they are the primary wage earners in the household [28]. Even when caregivers are secondary wage earners, their employment may take on greater importance if their patient (who was the primary wage earner) had to take a leave from the workforce while undergoing cancer treatment. Providing partial support for this idea, Hollenbeak et al. [29] found that the husbands of cancer survivors worked 1.5 more hours per week than the husbands of non-cancer survivors.

Nonetheless, it is important to note that the Veenstra et al. study [27] focused on spouses and partners of breast cancer patients. Breast cancer has a markedly favorable prognosis relative to most cancers and overwhelmingly affects women (many of whom have male partners). As such, findings may not be generalizable to other cancers or other types of caregivers (e.g., adult daughters, LGBTQ caregivers). Moreover, other studies suggest that a small number of caregivers (3–9%) do voluntarily quit their jobs, close their businesses, and pursue early retirement to devote more time and attention to their care recipients [15,26,30,31,32,33]. Unfortunately, the literature is unclear about whether caregivers who quit their jobs ever return to the labor market (e.g., after the patient dies or successfully completes cancer-directed treatment).

### 3.2. Work Modifications

Rather than exit the workforce entirely, 25–29% of caregivers make work modifications to satisfy their caregiving responsibilities [30,34]. These modifications include foregoing promotion or taking a less demanding job, changing from full- to part-time status, and changing work schedules (e.g., switching to the night shift, so they can take the care recipient to medical appointments) [15,34,35]. Estimates for work hour changes vary widely from 3 to 16 h per week [19,36]. Although some caregivers report taking on more work hours [30], most reduce their work hours, with the greatest reductions occurring during the terminal phase of the disease [27,31,32,36,37,38]. For some caregivers, a temporary reduction in work hours may precipitate the decision to change from full to part-time status [33,35].

Many caregivers take formal time off from work (paid or unpaid) to provide care. An analysis of 202 employed caregivers who were recruited from a population-based cohort of African American breast, colorectal, lung, and prostate cancer survivors found that more than half (52%) took paid time off from work to provide care, including 15% who took at least one month off [35]. However, in some workplaces, paid sick leave is uncommon and/or does not apply to the care of sick family members [34]. In addition, caregivers who have exhausted their allotted paid time off may have no recourse but to take unpaid time off. In fact, in that same population-based study, over a quarter of caregivers (27%) took unpaid time off, including 11% who took at least one month of unpaid time [35]. In the U.S., the Family and Medical Leave Act (FMLA) allows eligible employees to take up to 12 weeks of unpaid, protected leave annually for family and medical reasons with health insurance coverage continuation [39]. However, not all employees are covered under FMLA, and unpaid leave is financially undesirable for many.

### 3.3. Absenteeism

Absenteeism refers to any failure to report for or remain at work as scheduled, regardless of the reason. Studies from both the U.S. [15] and Canada [31] estimate that about half of employed caregivers come in late to work, leave work early, or take time off to accommodate their caregiving responsibilities. In fact, a large population-based study found that cancer caregivers are 1.75 times more likely to experience absenteeism relative to non-caregivers [40]. Reasons include transporting patients to medical appointments, caregiving activities and medical appointments taking longer than expected, and dealing with unanticipated issues related to patient care [30]. A U.S. study of 80 family caregivers of patients with primary malignant brain tumors found that one-third of employed caregivers experienced lost work hours due to providing care [41]. Although it is difficult to obtain precise estimates, studies have reported 11 lost work hours per week due to caregiving [42] to up to 7 days per month [43]. One study even estimated that cancer caregivers may lose as much as half of their workdays per month to assist with patient care [44].

### 3.4. Presenteeism

Presenteeism refers to reduced productivity while at work [45,46]. Overall productivity loss has been described as an “iceberg effect”, with the visible part of the iceberg representing absenteeism and the vast hidden area underneath as presenteeism [46]. Supporting this idea, a European study of lung cancer caregivers’ presenteeism, valued at $8676 per year, had a larger impact on the overall cost of the work impact of providing lung cancer care than absenteeism ($3234 per year) [47]. Population-based research suggests that cancer caregivers are 1.54 times more likely than non-caregivers to experience presenteeism [40], and the proportion of caregiver work productivity loss as a function of presenteeism has been estimated at 13–27% [42,48,49]. Studies have posited a variety of explanations including caregiver fatigue, worry, and time spent during the workday discussing patient care (e.g., with family, healthcare providers) [30], managing household responsibilities, and attending to patient medical needs (e.g., symptom management, transportation to medical appointments) [49,50]. Moreover, presenteeism may have downstream consequences for caregiver employment, with some caregivers reporting being overlooked for promotions due to their decreased availability [30], and others receiving warnings from their employers about their performance [15]. However, these long-term impacts are not fully understood.

### 3.5. Mental Health Impacts

While informal cancer caregiving is often an affordable and preferable alternative to paid caregiving, it can be an intensely burdensome and emotionally draining experience [7,31]. Cancer caregivers report a variety of mental health concerns including depression [51], social isolation [52], loss of self-identity [53], insomnia [54], and financial distress [55]. Over time, the caregiver’s mental health can wear on his/her physical health [56], adversely affect the patient’s mental health [57], and result in poor informal care quality [58]. Employed caregivers may also be a particularly vulnerable subgroup as over 64% report at least some difficulty balancing work and their caregiving responsibilities [35,59,60]. Studies have found that caregivers who are unable to complete work tasks experience greater job-related stress [30] and diminished perceptions of self-worth [61]. Caregivers with little or no flexibility in work hours also report greater stress than those with more flexible work arrangements [30].

With regard to depression, some studies describe higher levels of depressive symptoms among employed caregivers [62], whereas others have found no differences between employed and non-employed caregivers [49]. Still others have found that non-employed caregivers experience higher levels of burden than employed caregivers [15]. One explanation for this discrepancy is that even though balancing work and caregiving can be stressful, going to work affords a number of benefits including social support, respite, and economic security for caregivers [30,60,63,64]. It also provides a means for caregivers to preserve their individual identities (separate from the caregiving role) and foster a semblance of normalcy [61].

### 3.6. Vulnerable Caregivers

A variety of patient-related factors may increase likelihood of poor employment or mental health outcomes for informal cancer caregivers. For example, one study found that caregivers of colorectal (60%) and lung (54.2%) cancer patients reported more difficulty balancing work and caregiving than breast (34.4%) or prostate (29.5%) caregivers [35]. Caregivers of patients with newly diagnosed disease are more likely to report lost hours from work [34], and caregivers of patients who are undergoing chemotherapy or bone marrow transplant are more likely to take off work or make extended employment changes than caregivers of patients not undergoing these treatments [27,34,35]. Having a care recipient with advanced stage or terminal cancer, or more functional limitations, is associated with increased absenteeism [31,34,41,49], and having a care recipient in the terminal and palliative stages is associated with greater presenteeism [31]. With regard to sociodemographic factors, married/partnered caregivers are more likely to report absenteeism [35] and presenteeism [49] than unmarried caregivers. Female caregivers are more likely to report feeling exhausted and fatigued relative to male caregivers, and Hispanic caregivers are less likely to be employed relative to non-Hispanic white caregivers [27]. In the context of health equity and cancer disparities, more research could be done to examine potential risk/resilience factors for employed caregiver outcomes. Characteristics such as different employee segments (e.g., hourly workers, contractors, full-time, etc.) or employer industry (e.g., service, industrial, professional, etc.), among other attributes may exert unmeasured influence on caregiver vulnerability.

## 4. Research Agenda

Below (see also Table 1) we propose a broad research agenda that addresses knowledge gaps and methodological concerns that we have identified in this review. We also propose some health equity considerations to enhance generalizability and inform the development of resources and programs to support caregivers.

### 4.1. Knowledge Gaps

Critical knowledge gaps endure in our understanding of the effects of providing informal care to someone with cancer while being employed. Foremost among these, the range of resource and support needs of employed caregivers at different points along the cancer trajectory (e.g., at diagnosis, during treatment, terminal phase, post-treatment survivorship) must be ascertained so that more targeted programs, resource materials, and policies can be developed. The potential intersectionality between patient and caregiver factors should also be examined to elucidate which caregivers are at increased risk for poor outcomes (e.g., caregivers who are shift workers who are caring for patients with terminal cancer).

Given that effective and efficient initiatives that conserve scarce resources are likely to be implemented and sustained by employers, more research is needed to identify vulnerable subgroups of caregivers who may need additional supports and/or benefit from early intervention (e.g., at diagnosis). In a related vein, while some sociodemographic factors have been associated with increased risk for poor caregiver employment or mental health outcomes (i.e., gender, race, marital status), employment-related factors (e.g., size of employer, employment sector) should also be considered to guide the development of programs that are aligned to the unique issues faced by caregivers in different industries or employment settings.

The work impacts of caregiving warrant deeper exploration. For example, studies have demonstrated that absenteeism and presenteeism are issues for caregivers, but their downstream effects on caregiver career trajectories are relatively unknown. Developing greater understanding of the career-related impacts of cancer caregiving (e.g., impacts to work responsibilities, upward mobility, relationships with coworkers and managers, etc.) could greatly inform the development of workplace interventions to support caregivers. Workplace accommodations may provide partial relief to caregivers and help prevent them from making work modifications that could adversely impact their careers and livelihood. Discerning the role that different work accommodations (e.g., scheduling flexibility, unpaid or paid leave, remote work, etc.) may play in the prevention and/or reduction of work modifications, the cost/cost-effectiveness of these accommodations, and the barriers/facilitators of making them from the employer’s perspective requires further investigation.

Finally, studies should seek to clarify and reconcile the dichotomy between employed caregiver-specific burdens and the social and emotional benefits the workplace provides. Understanding caregiver characteristics that contribute to an overall positive or negative employment experience could also help to identify vulnerable caregivers who are at increased risk for poor mental health outcomes and develop more targeted programs and supports to meet their needs.

### 4.2. Methological Issues

Regarding methodological issues, qualitative interview studies could be extremely useful in describing caregiver needs and elucidating preferences for intervention. They could also be useful for clarifying employer attitudes, available resources, and potential barriers for implementation. At the same time, the reliance on small (*n* < 200) sample sizes, clinically recruited samples, and cross-sectional designs has affected the robustness of research findings. Studies that analyze large population-based datasets could improve generalizability and yield new insights. Prospective studies could elucidate how caregiver challenges and needs change and evolve across the extended treatment horizon to guide the timing of programs as well as resource allocation. Research is also needed to develop and test the feasibility, acceptability, and efficacy of healthcare interventions and employer and community-based programs to support employed caregivers. Toward this end, pragmatic trials that allow for flexibility in the delivery of caregiver interventions are needed. Researchers will also need to evaluate outcomes that are important to stakeholders, including cost, cost savings, and revenue generation potential in order to translate effective interventions from research to practice.

### 4.3. Health Equity Considerations

Given that the economic burdens and health disparities associated with informal caregiving are experienced across a number of identity-based strata, including race/ethnicity and socioeconomic status, more research is needed to investigate the challenges of employed caregivers through a health equity lens. For example, we know very little about the unique challenges of employed LGBTQ caregivers, older aged caregivers, or caregivers who are not a close family member of the patient (e.g., neighbors, close friends). In a similar vein, the role that health literacy and citizenship or immigration status may play in caregivers’ awareness and ability to navigate employment benefits and negotiate for accommodations is largely unknown. The interconnected nature of health and economic capacity also makes clear the need for a more multilevel approach to narrowing disparities that includes policy change, employer-based programs, and healthcare interventions. Specifically, research that examines support gaps for employed caregivers and multilevel factors (e.g., structural, environmental) that influence health disparities among historically marginalized caregiving communities can inform and shape current and future programs and policies on paid family and medical leave. Greater collaboration between research, healthcare, employers, and communities is also needed to discern what each entity can do to educate and link caregivers to available benefits and resources.

## 5. Practice Agenda

Below (see also Table 2), we propose some strategies that could be incorporated in programs to address the work and mental health impacts of cancer on employed caregivers that were identified in this review.

### 5.1. Work Accommodations

Employers may offer accommodations to cancer patients and survivors as a means of supporting them to work or return to the workforce. However, interventions to support survivors may not be entirely successful if they are not extended to also include their caregivers. The varied work-related impacts of cancer caregiving for employed caregivers suggest that more robust accommodation for caregivers should be considered to mitigate these impacts and their potential downstream effects. For example, to forestall decisions to exit the workforce, employers might implement return-to-work planning for caregivers who decide to take time off at the time of the patient’s diagnosis to ensure a successful return to the job. They might also consider outlining the process of a graduated return to work, where the caregiver may begin work part-time and gradually increase to full-time status [65].

### 5.2. Communication

Some caregivers may not be aware of the importance of communicating their needs to their employers, and others may benefit from guidance on how to advocate for themselves in this context. Therefore, cancer centers and community-based organizations can work to empower caregivers to start conversations with employers regarding the accommodations they may need.

As the demand for workplace programs to assist family caregivers is likely to increase as more workers assume caregiving responsibilities, it will be important for the employers and community-based organizations to serve as an information resource. Currently, most companies with programs to assist employed caregivers are large corporations, and only 25% of large businesses offer such programs [66]. Given that most Americans are employed by small businesses, few have access to caregiver assistance programs in the workplace, and healthcare and community-based organizations may need to step in and fill this gap. Even when services are available within the workplace, employee utilization rates are low due to organizational communication and education factors [67]. To increase utilization of caregiver assistance programs, employers need to clearly communicate what supports are available and encourage employees to read, understand, and access workplace programs. Managers should be informed about the benefits gained by employee participation to provide better support for employee attendance during work hours. Needs assessments should be conducted to direct programming and services, and employee programs should be evaluated regularly to ensure they continue to be utilized and are meeting caregiver needs.

### 5.3. Education

Research has underscored the importance of employers in mitigating burden for employed caregivers [68]. Therefore, training and education for human resources personnel about the unique impacts of cancer caregiving should be considered as well as how to offer successful assistance programs to meet the needs of employees. When properly trained, managers can open conversations about caregiving in a supportive fashion with their employees and help them to problem solve by connecting them to appropriate employment and community-based resources.

Another way to address the impacts of cancer caregiving is to support caregivers through education about workplace accommodation and understanding of roles among employers, insurers, the medical team, and patients. Employee benefits such as employee assistance programs, workplace or community support groups, family health insurance, and retirement benefits could also be points of education for caregivers that might assist with mitigating burden [15,27,30,41]. While a directory type resource could be a useful source of information, more interactive resources, such as free workshops, counseling, or classes could be explored as potentially more expedient methods toward this end.

Cancer centers and community-based organizations can also support caregivers seeking workplace accommodations by compiling resources and tools that outline relevant processes and policies. For example, a Canadian web-based resource for cancer patients (https://www.cancerandwork.ca/, accessed on 19 July 2022) that offers a variety of resources pertaining to workplace accommodations, return-to-work transition, etc., may offer a template for the development of similarly widely available and easily digestible resources that can be accessed by caregivers to assess needs, challenges, and supports related to employment.

### 5.4. Resources

Connecting employed caregivers to resources (e.g., social or financial support, accomodations, etc.) may help to empower and support them as they strive to balance work and caregiving responsibilities. Clinicians can help ease caregiver burden by providing experienced, specially trained patient navigators who are knowledgeable about available supports and can help families to make use of the appropriate resources [68]. These navigators can facilitate obtaining resources such as workplace accommodations for caregivers—for example, by providing medical documentation or by educating caregivers about available workplace protections. Community-based resources such as social services agencies could play a parallel role in assisting the employed caregiver. For instance, social service workers might consider integrating counseling sessions with the caregiver into their cancer patient support plan. These sessions could explicitly consider work-related topics, such as planning time off or determining how best to inform supervisors and coworkers about a loved one’s cancer diagnosis [30].

Screening could be used to identify vulnerable caregivers and connect them with resources based on identified unmet needs. Employed caregivers could be proactively screened for distress or burden within the healthcare system, and workers could put at-risk individuals in touch with professionals who may be able to offer services. CancerSupportSource^®^–Caregiver [69] is a validated electronic distress screening program that was designed to address the unique concerns of cancer caregivers [70,71], and it is being implemented at more than 40 Cancer Support Community facilities. The web-based tool asks caregivers to rate their level of concern for 33 problems related to their own self-care needs, emotional well-being, caregiving tasks, and perceived concerns about the patient’s well-being. If a concern is rated as low, caregivers can request information, but if they rate a concern as moderate or serious, they could request information, a referral, or both. The Department of Veterans Affairs provides another model for intervention wherein the clinical record shows caregiver responses to a set of screening questions about burden (Zarit Burden scale, short form) and a plan for meeting caregiver needs [68]. If screening responses indicate a need for psychosocial support, healthcare workers can liaise caregivers to provide healthcare and community resources.

## 6. Conclusions

It is our hope that this narrative review will facilitate a greater appreciation of the impact of cancer on employed caregivers and stimulate discussion for future research and practice. Overall, extant research suggests that cancer caregiving results in significant financial costs in terms of lost time and income. As a result of managing work and caregiving responsibilities, caregivers undergo unique work-related impacts, including decisions to exit the workforce, making work modifications, absenteeism, or presenteeism. They also experience mental health impacts including depression, social isolation, and loss of self-identity. Prospective and population-based studies are needed to identify caregivers who are most at risk for negative employment and mental health outcomes. Qualitative studies could also elucidate the range of caregiver unmet needs and preferences for intervention. This additional information might contribute to a more complete model of caregiving stress among working adults and further processes for identifying vulnerable caregivers. Such models may encompass providing workplace accommodations such as return-to-work planning, training and education to empower caregivers to advocate for their needs, and connecting caregivers to community resources to fulfill unmet needs. Ultimately, a multipronged effort on behalf of employers, healthcare, and community stakeholders will likely be needed to address the varied work-related impacts of cancer caregiving.

## Figures and Tables

**Table 1:** Research Agenda.

Topic Area	Recommendations
Knowledge Gaps	● Ascertain caregiver needs at different timepoints along the cancer care continuum (e.g., at diagnosis, during treatment, terminal phase, post-treatment survivorship)
	● Identify caregivers who may need additional supports and/or benefit from early intervention
	● Develop clearer understanding of how work life is affected for different caregiver employee segments (e.g., hourly workers, professionals, etc.)
	● Explore potential intersectionality between patient and caregiver factors to elucidate caregivers at increased risk for poor outcomes
	● Clarify downstream effects of absenteeism and presenteeism
	● Develop clearer understanding of employment-related factors that impair the ability of caregivers to fulfill their role
	● Understand what accommodations are effective in preventing labor market withdrawal and the cost, cost-effectiveness, and barriers/facilitators of those accommodations
Methodological Issues	● Conduct qualitative studies to understand caregiver needs ● Conduct population-based studies to identify risk factors
	● Conduct longitudinal studies to understand how needs change over time ● Develop interventions and examine feasibility, acceptability, and efficacy
	● Discern metrics that are important to key stakeholders
Health Equity Considerations	● Investigate challenges of employed caregivers through a health equity lens
	● Develop clearer understanding of the unique challenges of LGBTQ caregivers, older aged caregivers, non-family caregivers, etc.
	● Investigate role that health literacy and citizenship/ immigration status play in caregivers’ ability to navigate employment benefits and negotiate for accommodations
	● Examine support gaps for employed caregivers and multilevel factors (e.g., structural, environmental) that influence health disparities among historically marginalized caregiving communities

**Table 2:** Practice Agenda.

Topic Area	Recommendations
Accommodations	● Provide robust accommodations (e.g., flexible work schedules, telecommuting options)
	● Help caregivers to identify and anticipate work-related needs and desired supports ● Include caregivers in programs designed to support cancer survivors in returning to work ● Provide return-to-work planning
Communication	● Empower caregivers to communicate with employers
	● Employers should clearly communicate what programs and supports are available for caregivers ● Employers should obtain employee input on caregiver assistance programs through needs assessments and program evaluations
Education	● Educate caregivers about their rights and leave policies
	● Develop resources/tools to map out processes and policies for workplace accommodations and returning to work ● Educate managers about cancer, accommodation, and work challenges of caregivers
Resources	● Patient navigators in healthcare systems may be helpful in connecting patients to healthcare and community resources ● Proactive screening can be used to identify vulnerable caregivers and connect them with community-based resources such as psychosocial support and/or integrated caregiver counseling sessions with cancer patient support plans
